# Supplementary material for: Investigation of Biomedical Students’ Knowledge on Glaucoma Reveals a Need for Education: A Cross-Sectional Study
Source: Healthcare (Basel). 2022 Jul 3;10(7):1241. doi: 10.3390/healthcare10071241 (PMC9319733; doi:10.3390/healthcare10071241)
Supplement: Supplementary file 1 [file healthcare-10-01241-s001.zip › healthcare-1756412-supplementary.pdf]

This questionnaire is used to determine attitudes and knowledge on glaucoma among biomedical students. Your engagement is completely anonymous and voluntary. By completing this questionnaire, you are providing a consent to take part in the study. Thank you for your time.

DEMOGRAPHIC CHARACHERISTICS

STUDY PROGRAM: MEDICINE DENTAL MEDICINE PHARMACY

GENDER: M F

AGE: ___

STUDY YEAR: 1 2 3 4 5 6

OPHTALMOLOGY COURSE GRADE 0 (NOT COMPLETED) 2 3 4 5

FAMILY HISTORY OF GLAUCOMA: YES NO DO NOT KNOW

ARE YOU GLAUCOMA PATIENT: YES NO DO NOT KNOW

SOURCES OF INFORMATION ON GLAUCOMA: Physician Friends or family Internet Print media Educational Materials

Q1. The most common cause of irreversible blindness is:

1. Cataract
2. Glaucoma
3. Age-dependent macular degeneration
4. Diabetic retinopathy
5. I don't know

Q2. Glaucoma can cause blindness. Glaucoma blindness is:

1. Reversible with all forms of therapy (surgery and medication)
2. Reversible only after surgery
3. Non-refundable
4. I don't know

Q3. The most common type of glaucoma is:

1. Primary open-angle glaucoma
2. Primary closed-angle glaucoma
3. Congenital glaucoma
4. Secondary glaucoma
5. I don't know

Q4. The causes of primary open-angle glaucoma are:

1. Hereditary factors
2. Elevated intraocular pressure
3. Infection
4. I don't know

Q5. Please indicate the main risk factors for primary open-angle glaucoma (multiple answers):

1. Elevated intraocular pressure
2. Elevated arterial pressure
3. Diabetes
4. Neurological diseases
5. Young age
6. Hyperopia (farsightedness)
7. Myopia (nearsightedness)
8. Positive family history of glaucoma
9. Eye trauma
10. Use of corticosteroids
11. Black race

Q6. Please indicate the most common signs and symptoms for primary open-angle glaucoma:

1. Sudden loss of central visual field
2. Sore eye
3. Red eye
4. Increased tearing of the eye
5. Periocular headache
6. Frontal headache
7. Photophobia
8. The disease is mostly asymptomatic
9. I don't know

Q7. Major diagnostic tests for glaucoma include (multiple answers):

1. Examination of visual acuity
2. Examination of the ocular background
3. Measurement of intraocular pressure (tonometry)
4. Visual field examination (perimetry)
5. Magnetic resonance imaging
6. I don't know

Q8. Glaucoma can be treated (multiple answers):

1. Eye drops
2. Surgery
3. Laser
4. There is no appropriate therapy
5. I don't know

Q9. Successful glaucoma treatment outcome is:

1. Healing

2. Disease control and prevention of blindness

3. Alleviation of symptoms, although the disease inevitably causes blindness

4. There is no appropriate therapy

5. I don't know

Q10. Pharmacological treatment of glaucoma lasts:

1. For weeks

2. For months

3. For years

4. For the rest of your life

5. I don't know

Q11. Do you know of any medication used to treat glaucoma**:**

1. NO

2. YES

Q12. Do you think that the knowledge you gained in college about glaucoma is enough?

1. YES

2. NO

Q13. When was the last time you had an eye examination?

1. 1 year ago (or less)

2. Two years ago

3. A year ago (and more)

Q14. If you suffer from glaucoma, what would be your attitude towards treatment:

1. Visit an ophthalmologist regularly and take prescribed medications without interruption

2. Sometimes visit an ophthalmologist and sometimes skip medication

3. Choose surgery to avoid taking drugs for life

Q15. In case surgery is your best treatment option, what would be your attitude:

1. Ready to agree to surgery

2. Try to delay surgery and continue to use medication

3. Use medication and try alternative therapies
